# SARS-COV-2 Presenting as New Onset Atrial Fibrillation: A Case Report

**DOI:** 10.7759/cureus.8054

**Published:** 2020-05-11

**Authors:** Jason Harhay, Muniba Khan, Shalin Shah, Amit Malhotra

**Affiliations:** 1 Internal Medicine, Buffalo General Medical Center, University at Buffalo, Buffalo, USA; 2 Internal Medicine, University at Buffalo, Buffalo, USA

**Keywords:** sars-cov-2, covid 19, atrial fibrillation, altered mental status

## Abstract

Current literature has documented numerous different presentations of SARS-COV-2 (COVID-19). Common symptoms include fever, cough and shortness of breath, however, lack of these symptoms does not exclude COVID-19. Given the incomplete understanding of the virus at this time, healthcare professionals must continue to remain informed of the vast number of clinical presentations of the virus to ensure early supportive treatment, ideally leading to improved outcomes.

## Introduction

The typical presentation of SARS-COV-2 (COVID-19) is fever, cough, tachypnea and shortness of breath [1]. However, with time, many other presentations have been documented, likely owing to an incomplete understanding of this new pathogen. To help inform the growing knowledge base, we present a patient with no documented past medical history who presented with new onset atrial fibrillation, pulmonary edema, and altered mental status.

The primary objective of this case report is to present an atypical presentation of the SARS-COV-2 virus.

## Case presentation

**Figure 1:**
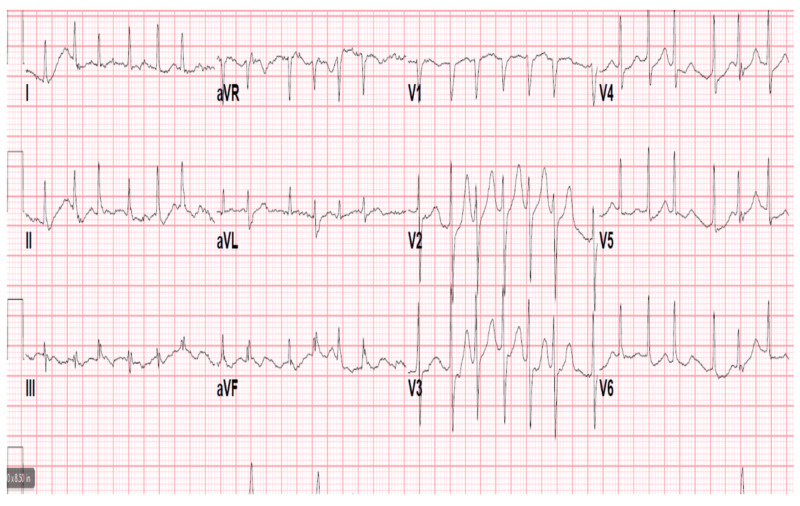
Atrial Fibrillation with Rapid Ventricular Response

**Figure 2:**
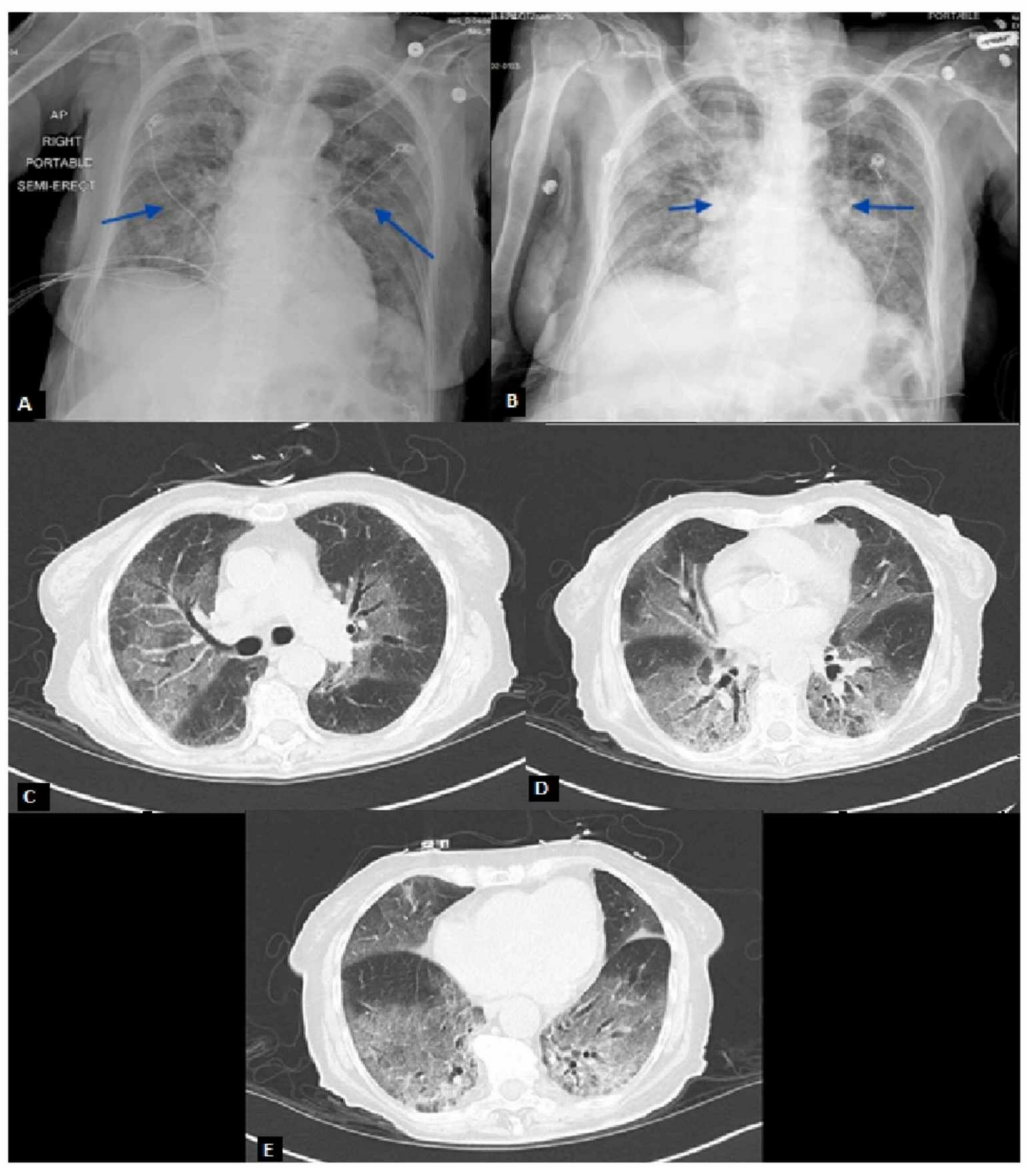
Chest X-Rays and CT Scan Demonstrating Bilateral Pulmonary Infiltrates (A) Hazy appearance of the lung fields with scattered airspace opacities. (B) Bilateral faint patchy airspace opacities slightly increased compared to initial imaging in Figure 2A. (C-E) Patchy ground-glass attenuation and smooth interlobular septal thickening involving all lobes, most pronounced within the right upper and bilateral lower lobes.

A 90-year-old African American female with no documented past medical history presented to the emergency department following a welfare check from Adult Protective Services. The patient was noted to have altered mentation by emergency medical services (EMS). In the emergency room, vitals demonstrated a temperature of 97.9, blood pressure of 141/78, heart rate of 140, and a respiratory rate of 24 not requiring oxygen supplementation. Physical exam was notable for tachycardia with irregularly irregular rhythm and mild bibasilar crackles with no jugular venous distention or lower extremity edema. The patient was found to be in atrial fibrillation with a rapid ventricular response (afib with RVR) on initial EKG (Figure 1). Chest X-ray revealed diffuse pulmonary edema and hazy opacities (Figure 2A). Repeat chest X-ray following diuresis demonstrated bilateral interstitial infiltrates (Figure 2B). CT scan of the chest without IV contrast demonstrated multiple regions of ground-glass opacities (Figure 2C-2E). Labs revealed a sodium of 147, anion gap of 23, lactic acid level of 4.3, and pro-B-natriuretic peptide was 1637. Initial venous blood gas (VBG) demonstrated pH of 7.47 with pCO2 of 34. Additionally, troponins were negative over three blood draws. An echocardiogram revealed an ejection fraction of 65-70% with grade 1 diastolic dysfunction.

Given the patient’s imaging, COVID-19 polymerase chain reaction (PCR) testing was performed with a positive result on day three of hospitalization. That same day, the patient rapidly deteriorated and became hypoxic prompting intubation and vasopressor support. The patient was treated with supportive care in the intensive care unit (ICU) and was started on hydroxychloroquine and azithromycin. She was extubated on day nine of admission.

## Discussion

Since the onset of COVID-19, a number of presentations have been documented. The typical presentation includes symptoms of fever, cough, and shortness of breath. However, these symptoms are not always seen on the initial presentation. As a result, suspicion for this viral infection may erroneously become lower on the differential diagnosis. As noted in our case presentation, the patient presented with altered mental status and afib with RVR. A study published in “Heart Rhythm” suggested that atrial fibrillation can be induced by a systemic inflammatory response and increased sympathetic tone [2]. The study focused on influenza; however, other viral and bacterial infections can result in atrial fibrillation, particularly in sepsis patients.

Sinus tachycardia (the most common arrhythmia in COVID-19 patients to date), myocardial injury, hypotension, bradycardia, and transient cardiomegaly have been documented as complications of SARS, particularly in patients with underlying cardiovascular disease [3-5]. Current literature has proposed the viral involvement of cardiomyocytes and systemic inflammation as the mechanism of myocardial injury [4]. Few other explanations have been provided for cardiovascular involvement. It is possible that the proposed systemic inflammation involved in myocardial injury induces increased sympathetic tone which would provide an explanation as to why some COVID-19 patients develop arrhythmias.

## Conclusions

New manifestations of COVID-19 are being documented on a daily basis. Our patient's initial presentation resulted in decreased initial suspicion for COVID-19 infection given the lack of typical signs and symptoms. As the literature grows, earlier testing and diagnosis will follow ultimately leading to better outcomes. Further research and data collection will be necessary to develop a better understanding of the underlying mechanisms and clinical outcomes involved with the manifestations of COVID-19.
